# Risk factors for HPV infection and high-grade cervical disease in sexually active Japanese women

**DOI:** 10.1038/s41598-021-82354-6

**Published:** 2021-02-03

**Authors:** Manako Yamaguchi, Masayuki Sekine, Sharon J. B. Hanley, Risa Kudo, Megumi Hara, Sosuke Adachi, Yutaka Ueda, Etsuko Miyagi, Takayuki Enomoto

**Affiliations:** 1 Department of Obstetrics and Gynecology, Niigata University Graduate School of Medical and Dental Sciences, 1-757 Asahimachi-dori, Chuo-ward, Niigata, 951-8510 Japan; 2 Department of Obstetrics and Gynecology, Hokkaido University Graduate School of Medicine, Sapporo, Japan; 3 Department of Preventive Medicine, Faculty of Medicine, Saga University, Saga, Japan; 4 Departments of Obstetrics and Gynecology, Osaka University Graduate School of Medicine, Suita, Japan; 5 Department of Obstetrics and Gynecology, Yokohama City University School of Medicine, Yokohama, Japan

**Keywords:** Cancer prevention, Gynaecological cancer, Disease prevention

## Abstract

In Japan, recommendations for HPV vaccines were suspended in 2013 due to unfounded safety fears. Although vaccine opponents claim modifying sexual behavior can prevent cervical cancer, no comprehensive data exist on sexual behavior and the risk of high-grade cervical disease in a Japanese population. This study investigates sexual behavior and the risk of HPV infection and cervical disease in 3968 women aged 20–41 yrs undergoing cervical screening between April 2014 and March 2016. Mean age at first intercourse was 18.4 yrs ± 2.8 and 32% of women reported ≥ 6 lifetime sexual partners. In regression analyses, number of partners was a significant risk factor for HPV infection. However, for high-grade disease (CIN2+), when HPV genotype was adjusted for, number of partners was not statistically significant. The greatest risk factor was an HPV16/18 infection (adjusted odds ratio 113.7, 95% CI: 40.8–316.9). In conclusion, we found that having an HPV16/18 infection and not sexual behavior was the most significant risk factor for high grade cervical disease in young Japanese women. These infections can be prevented by a highly effective vaccine and we recommend that the Japanese government resume proactive recommendations for the HPV vaccine immediately.

## Introduction

Cervical cancer is highly preventable and if diagnosed and treated early, highly curable. Yet it remains the fourth most frequent cancer in women globally. Almost all cases are caused by persistent infection with one or more sexually transmitted high-risk human papillomavirus (hrHPV) type^1,2^. Risk factors for HPV infection include: early onset of sexual activity, multiple sexual partners, or partners with multiple sexual partners^3,4^. Risk factors for viral persistence and progression to high grade cervical precancers and invasive cervical cancer include exogenous factors such as tobacco smoking, high parity and coinfection with other sexually transmitted infections^2,5^; viral factors such as HPV genotype, particularly HPV 16 and HPV 18, viral load and HPV variants^6,7^; and host cofactors such as factors related to immune response^2,8^.

Following the global call to action for the elimination of cervical by the World Health Organization’ s (WHO) Director General in 2018, a global strategy to accelerate cervical cancer elimination was drafted with clear targets for the period 2020–2030. These include that 80–100% of girls get vaccinated against HPV by the age of 15 yrs^9^. Therefore, HPV vaccination is an important public health intervention to prevent cervical cancer by routinely targeting adolescent girls before they become sexually active.

In Japan, there are over 10,000 cases of cervical cancer annually and more than 2700 women die from this disease^10^. Incidence of cervical cancer and high-grade cervical intraepithelial neoplasia (CIN) is increasing in women of reproductive age^11^. Biennial screening with cytology is recommended for women aged ≥ 20 yrs as secondary prevention^12^. However, screening is opportunistic and coverage is lower than most OECD countries (42.4% in women 20–69 yrs)^13^. Bivalent and quadrivalent HPV vaccines were approved in Japan in October 2009 and July 2011, respectively. Public funding became available from 2010 and from April 2013, the HPV vaccine was included in the National Immunization Program (NIP) for girls aged 12–16 yrs. However, after unfounded sensational reports of so-called ‘adverse events’ were published by the Japanese media and pressure by a ‘Victims’ Support Group, the Japanese Ministry of Health, Labour, and Welfare (MHLW) suspended proactive recommendations for the HPV vaccine in June 2013^14,15^. After that, coverage dramatically decreased, from > 70% in those born between 1994–1999 to < 1% in those born in 2000 and later^16^. The national education materials for schoolchildren recommend cancer screening for cervical cancer prevention in Japan, however, HPV vaccination are not described in the materials^17^.

One reason often given by groups opposing the HPV vaccine is that only women who engage in ‘risky’ sexual behavior are at risk for cervical cancer and by modifying behavior cervical cancer can be prevented.

Since there is a close correlation between sexual activity, HPV infection, and cervical cancer risk, but almost no comprehensive data that investigates a combination of all these factors in a Japanese population. We investigated the correlation between sexual behavior (age at sexual debut and number of sexual partners), HPV infection, including genotype-specific infection and high-grade cervical disease in sexually active Japanese women of reproductive age for a better comprehensive approach to cervical cancer prevention in Japan.

## Results

In total, 3968 women were enrolled in the study when attending for publicly funded cervical cancer screening in Niigata city between April 2014 and March 2016. Of those, 188 women were excluded due to age, duplicate results, missing results for HPV testing or equivocal HPV test results, leaving a total of 3780 women enrolled. The response rate to the questionnaire on sexual history was 87.4% (3304/3780). Seventy-three women were excluded from the analysis because they reported no previous sexual activity. Therefore, 3231 sexually active women were included in the final analyses.

### Background of participants (Table 1)

**Table 1:** Background of the participants.

	All		Group A (20–30 yrs)		Group B (35–36 yrs)		Group C (40–41 yrs)	
			Birth year: 1984–1995		Birth year: 1978–1980		Birth year: 1973–1975	
	(n = 3231)		(n = 2179)		(n = 725)		(n = 327)	
	n	%	n	%	n	%	n	%
Age								
Mean(± SD)	29.8	(± 5.7)	26.3	(± 2.9)	35.5	(± 0.5)	40.3	(± 0.5)
Age at first intercourse (yrs)								
≤ 14	109	3.4%	91	4.2%	16	2.2%	2	0.6%
15–16	779	24.1%	638	29.3%	114	15.7%	27	8.3%
17–19	1441	44.6%	907	41.6%	361	49.8%	173	52.9%
≥ 20	902	27.9%	543	24.9%	234	32.3%	125	38.2%
Mean(± SD)	18.4	(± 2.8)	18.0	(± 2.7)	18.8	(± 2.9)	19.6	(± 3.3)
Number of lifetime sexual partners								
1	526	16.3%	400	18.4%	77	10.6%	49	15.0%
2–5	1670	51.7%	1097	50.3%	383	52.8%	190	58.1%
6–9	580	18.0%	372	17.1%	148	20.4%	60	18.3%
≥ 10	455	14.1%	310	14.2%	117	16.1%	28	8.6%
Smoking history								
Never	2290	70.9%	1592	73.1%	477	65.8%	221	67.6%
Former	475	14.7%	271	12.5%	141	19.6%	63	19.4%
Current	444	13.7%	302	13.9%	101	13.9%	41	12.5%
Missing	52	1.6%	32	1.5%	17	2.3%	3	0.9%
HPV vaccination^a^								
Vaccinated^b^	112	3.5%	94	4.3%	15	2.1%	3	0.9%
Unvaccinated	3116	96.4%	2083	95.6%	709	97.8%	324	99.1%
Missing	3	0.1%	2	0.1%	1	0.1%	0	0.0%

^a^Self-reported.

^b^Bivalent or quadrivalent.

**Figure 1:**
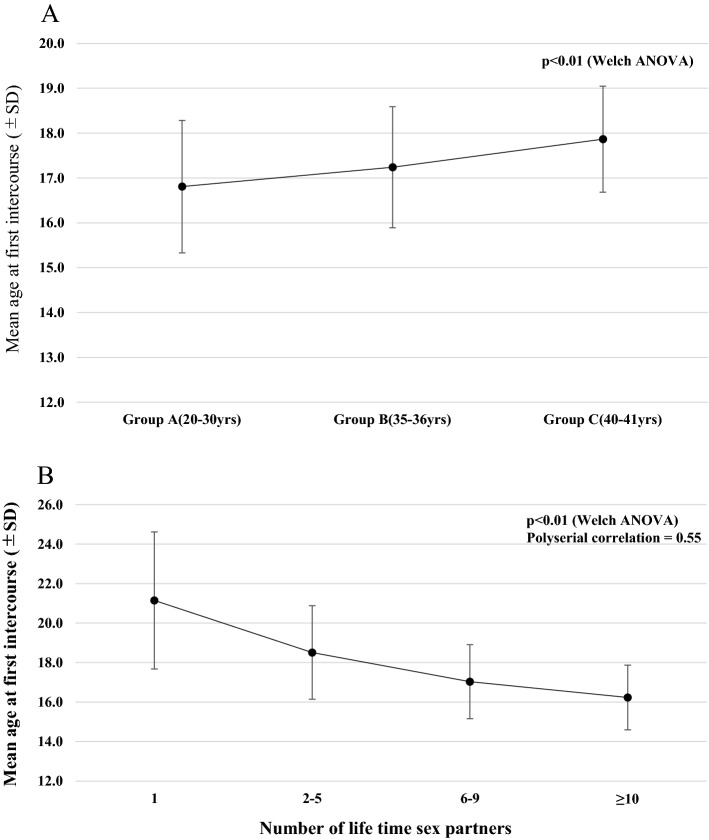
(**A**) Comparison between age-groups of mean age at sexual debut in women who first had sex as a teenage (n = 2329) Mean age at first intercourse was significantly earlier in group A (16.8 ± 1.5), compared to groups B (17.2 ± 1.4) and C (17.9 ± 1.2) (*p* < 0.01). (**B**) Correlation between multiple number of lifetime sexual partners and lower age at first intercourse (n = 3231). There was a significant correlation between lifetime number of sexual partners and lower age at first intercourse (*p* < 0.01, polyserial correlation = 0.55).

Number of participants in each age-group was 2179, 725 and 327 for groups A (20–30 yrs), B (35–36 yrs) and C (40–41 yrs), respectively. Mean overall age of participants was 29.8 yrs ± 5.7 and 26.3 yrs ± 2.9 in group A. Mean overall age at first intercourse was 18.4 yrs ± 2.8 yrs. For age at first intercourse, 3.4%, 24.1%, 44.6% and 27.9% of women had their first sexual experience at 14 yrs or younger, 15–16 yrs, 17–19 yrs or ≥ 20, respectively, which indicates 72.1% of women (2329/3231) became sexually active as a teenager. Among these women, mean age at first intercourse was significantly earlier in group A (16.8 ± 1.5), compared to groups B (17.2 ± 1.4) and C (17.9 ± 1.2) (*p* < 0.01, Fig. 1A). Around half of participants (n = 1670, 51.7%) had 2–5 sexual partners and approximately 30% of participants had ≥ 6 sex lifetime partners. There was a significant correlation between lifetime number of sexual partners and lower age at first intercourse (*p* < 0.01, polyserial correlation = 0.55, Fig. 1B). History of smoking (former or current) was 28.4% for all participants. Only 112 (3.5%) women reported having the HPV vaccine, mostly because they were born before 1994 and not eligible for free vaccination.

### Prevalence of HPV infection, abnormal cervical cytology and histology (Table 2)

**Table 2:** Prevalence of HPV infection, abnormal cervical cytology and histology.

	All		Group A (20–30 yrs)		Group B (35–36 yrs)		Group C (40–41 yrs)		*P* for trend
	(n = 3231)		(n = 2179)		(n = 725)		(n = 327)		
	n	%	n	%	n	%	n	%	
HPV infection									
High-risk HPV^a^	400	12.4%	331	15.2%	50	6.9%	19	5.8%	< 0.01
HPV16/18	76	2.4%	66	3.0%	7	1.0%	3	0.9%	< 0.01
HPV16/18/31/33/45/52/58	231	7.1%	195	8.9%	26	3.6%	10	3.1%	< 0.01
Cytology									
NILM	3033	93.9%	2021	92.7%	692	95.4%	320	97.9%	–
ASC-US or worse	198	6.1%	158	7.3%	33	4.6%	7	2.1%	< 0.01
Histology									
CIN1	54	1.7%	43	2.0%	9	1.2%	2	0.6%	0.04
CIN2	33	1.0%	29	1.3%	4	0.6%	0	0.0%	0.01
CIN3	13	0.4%	7	0.3%	5	0.7%	1	0.3%	0.60
ICC	1	0.0%	1	0.0%	0	0.0%	0	0.0%	0.99

^a^HPV16/18/31/33/35/39/45/51/52/56/58/59/68 (HC2).

In group A, 15.2% of women had a hrHPV infection. This was significantly higher than in groups B and C, at 6.9% and 5.8%, respectively, (*p* for trend < 0.01). A similar trend was observed for HPV 16/18 genotypes (*p* for trend < 0.01) and HPV16/18/31/33/45/52/58 (*p* for trend < 0.01) respectively. Prevalence of HPV type-specific infections is shown in supplementary Table 1. Prevalence of abnormal cytology [atypical squamous cells of undetermined significance (ASC-US) or worse] was significantly higher in group A (7.3%) compared to groups B (4.6%) and C (2.1%), *p* for trend < 0.01. Regarding histology, for CIN 1 and CIN2, prevalence decreased with age; *p* for trend was 0.04 and 0.01, respectively. For CIN3, there was no significant trend. Prevalence was highest in women 35–36 yrs, after which levels in woman 40–41 yrs decreased to levels similar to those in woman aged 20–30 yrs. There was one invasive cervical cancer in a woman aged 29 yrs.

### Correlation between age at first intercourse, rates of HPV infection and high-grade cervical disease (Table 3)

**Table 3:** Correlation between age at first intercourse rates of HPV infection and high-grade cervical disease (CIN2 or worse).

Age at first intercourse		n	(%pos)	Univariate			Multivariate								
							Model 1			Model 2			Model 3		
				OR	(95% CI)	*P* value	aOR	(95% CI)	*P* value	aOR	(95% CI)	*P* value	aOR	95% (CI)	*P* value
High-risk HPV^a^	≤ 14	29/109	(26.6)	Reference		–	Reference		–	Reference		–	Reference		–
	15–16	128/779	(16.4)	0.5	(0.3–0.9)	0.01	0.6	(0.4–0.9)	0.02	0.7	(0.4–1.1)	0.09	0.8	(0.5–1.4)	0.46
	17–19	176/1441	(12.2)	0.4	(0.2–0.6)	< 0.01	0.5	(0.3–0.7)	< 0.01	0.6	(0.4–1.0)	0.04	1.1	(0.7–1.8)	0.77
	≥ 20	67/902	(7.4)	0.2	(0.1–0.4)	< 0.01	0.3	(0.2–0.5)	< 0.01	0.4	(0.2–0.7)	< 0.01	1.2	(0.7–2.1)	0.54
	*P* for trend					< 0.01			< 0.01			< 0.01			0.09
HPV16/18	≤ 14	5/109	(4.6)	Reference		–	Reference		–	Reference		–	Reference		–
	15–16	23/779	(3.0)	0.6	(0.2–1.6)	0.30	0.6	(0.2–1.7)	0.38	0.7	(0.3–1.9)	0.51	1.0	(0.4–2.8)	0.99
	17–19	36/1441	(2.5)	0.5	(0.2–1.3)	0.13	0.6	(0.2–1.6)	0.29	0.7	(0.3–2.0)	0.56	1.8	(0.7–5.1)	0.24
	≥ 20	12/902	(1.3)	0.2	(0.1–0.7)	0.01	0.3	(0.1–0.9)	0.04	0.4	(0.1–1.3)	0.15	2.6	(0.8–8.6)	0.11
	*P* for trend					< 0.01			0.03			0.16			0.01
HPV16/18/31/33/45/52/58	≤ 14	15/109	(13.8)	Reference		–	Reference		–	Reference		–	Reference		–
	15–16	78/779	(10.0)	0.7	(0.4–1.2)	0.19	0.7	(0.4–1.3)	0.30	0.9	(0.5–1.6)	0.60	1.1	(0.6–2.1)	0.75
	17–19	102/1441	(7.1)	0.5	(0.3–0.8)	0.01	0.6	(0.3–1.0)	0.06	0.8	(0.4–1.4)	0.40	1.5	(0.8–2.9)	0.20
	≥ 20	36/902	(4.0)	0.2	(0.1–0.5)	< 0.01	0.3	(0.2–0.6)	< 0.01	0.5	(0.2–1.0)	0.04	1.9	(0.9–3.9)	0.10
	*P* for trend					< 0.01			< 0.01			0.01			0.02
CIN2 or worse	≤ 14	5/109	(4.6)	Reference		–	Reference		–	Reference		–	Reference		–
	15–16	17/779	(2.2)	0.4	(0.2–1.2)	0.09	0.4	(0.2–1.2)	0.10	0.5	(0.2–1.3)	0.15	0.6	(0.2–1.7)	0.32
	17–19	19/1441	(1.3)	0.3	(0.1–0.7)	0.01	0.3	(0.1–0.8)	0.01	0.3	(0.1–1.0)	0.05	0.6	(0.2–1.9)	0.42
	≥ 20	6/902	(0.7)	0.1	(0.0–0.4)	< 0.01	0.1	(0.0–1.5)	< 0.01	0.2	(0.1–0.7)	0.01	0.7	(0.2–2.7)	0.59
	*P* for trend					< 0.01			< 0.01			0.01			0.80

*CIN* cervical intraepithelial neoplasia, *OR* odds ratio, *aOR* adjusted odds ratio.

*Model 1* adjusted for age.

*Model 2* adjusted for age and smoking history.

*Model 3* adjusted for age, smoking history and number of lifetime sexual partners.

^a^HPV16/18/31/33/35/39/45/51/52/56/58/59/68 (HC2).

Prevalence of hrHPV peaked in women who became sexually active at 14 yrs or younger (26.6%), followed by a gradual decline in prevalence at 16.4%, 12.2% and 7.4% in women whose sexual debut was at 15–16 yrs, 17–19 yrs and ≥ 20 yrs, respectively (*p* for trend < 0.01). Statistical significance remained after adjusting for age and smoking history (model 2), but the trend was lost after making an additional adjustment for number of sexual partners (model 3). A similarly trend was seen for the prevalence of HPV16/18, HPV16/18/31/33/45/52/58 and CIN2 or worse (CIN2+) (*p* for trend < 0.01). Prevalence of CIN2+ was highest in women who became sexually active at 14 yrs or younger (4.6%), followed by a gradual decline in prevalence at 2.2%, 1.3% and 0.7% in the women whose sexual debut was at 15–16 yrs, 17–19 yrs and > 20 yrs, respectively (*p* for trend < 0.01). Statistical significance remained after adjusting for age and smoking history (model 2), but the trend was lost after further adjustment for number of sexual partners (model 3).

### Correlation between number of lifetime sexual partners, rates of HPV infection and abnormal histology (Table 4)

**Table 4:** Correlation between number of lifetime sexual partners, rates of HPV infection and high-grade cervical disease (CIN2 or worse).

Number of lifetime sexual partners		n	(%pos)	Univariate			Multivariate								
							Model 1			Model 2			Model 3		
				OR	(95% CI)	*P* value	aOR	(95% CI)	*P* value	aOR	(95% CI)	*P* value	aOR	(95% CI)	*P* value
High-risk HPV^a^	1	19/526	(3.6)	Reference		–	Reference		–	Reference		–	Reference		–
	2–5	150/1670	(9.0)	2.6	(1.6–4.3)	< 0.01	3.1	(1.9–5.0)	< 0.01	3.0	(1.8–4.9)	< 0.01	3.3	(2.0–5.4)	< 0.01
	6–9	117/580	(20.2)	6.7	(4.1–11.1)	< 0.01	8.5	(5.1–14.1)	< 0.01	8.0	(4.8–13.4)	< 0.01	9.3	(5.4–16.1)	< 0.01
	≥ 10	114/455	(25.1)	8.9	(5.4–14.8)	< 0.01	10.4	(6.3–17.4)	< 0.01	9.4	(5.5–16.0)	< 0.01	11.3	(6.4–19.9)	< 0.01
	*P* for trend					< 0.01			< 0.01			< 0.01			< 0.01
HPV16/18	1	1/526	(0.2)	Reference		–	Reference		–	Reference		–	Reference		–
	2–5	18/1670	(1.1)	6.0	(0.8–44.8)	0.08	7.1	(0.9–53.3)	0.06	7.3	(1.0–55.0)	0.05	9.1	(1.2–69.3)	0.03
	6–9	33/580	(5.7)	35.3	(4.8–259.2)	< 0.01	46.8	(6.3–345.2)	< 0.01	50.2	(6.8–373.3)	< 0.01	77.6	(10.0–600.0)	< 0.01
	≥ 10	24/455	(5.3)	34.5	(4.7–256.4)	< 0.01	42.1	(5.7–313.6)	< 0.01	46.6	(6.1–354.2)	< 0.01	78.7	(9.9–628.2)	< 0.01
	*P* for trend					< 0.01			< 0.01			< 0.01			< 0.01
HPV16/18/31/33/45/52/58	1	5/526	(1.0)	Reference		–	Reference		–	Reference		–	Reference		–
	2–5	82/1670	(4.9)	5.4	(2.2–13.5)	< 0.01	6.4	(2.6–16.0)	< 0.01	6.3	(2.5–15.7)	< 0.01	7.2	(2.9–18.1)	< 0.01
	6–9	70/580	(12.1)	15.0	(6.0–37.4)	< 0.01	19.5	(7.8–49.0)	< 0.01	18.5	(7.3–46.9)	< 0.01	23.6	(9.0–61.5)	< 0.01
	≥ 10	74/455	(16.3)	21.3	(8.5–53.2)	< 0.01	25.6	(10.2–64.1)	< 0.01	23.3	(9.1–59.4)	< 0.01	31.4	(11.8–83.3)	< 0.01
	*P* for trend					< 0.01			< 0.01			< 0.01			< 0.01
CIN2 or worse	1	0/526	(0.0)	0.0		0.99	0.0		0.99	0.0		0.99	0.0		0.99
	2–5	16/1670	(1.0)	Reference		–	Reference		–	Reference		–	Reference		-
	6–9	13/580	(2.2)	2.4	(1.1–5.0)	0.02	2.5	(1.2–5.2)	0.02	2.4	(1.1–5.1)	0.02	2.4	(1.1–5.4)	0.03
	≥ 10	18/455	(4.0)	4.4	(2.2–8.8)	< 0.01	4.4	(2.2–8.7)	< 0.01	4.2	(2.0–8.8)	< 0.01	4.1	(1.9–9.2)	< 0.01
	*P* for trend					< 0.01			< 0.01			< 0.01			< 0.01

*CIN* cervical intraepithelial neoplasia, *OR* odds ratio, *aOR* adjusted odds ratio.

*Model 1* adjusted for age.

*Model 2* adjusted for age and smoking history.

*Model 3* adjusted for age, smoking history and age at first intercourse.

^a^HPV16/18/31/33/35/39/45/51/52/56/58/59/68 (HC2).

Prevalence of hrHPV peaked in women who had ≥ 10 lifetime sexual partners (25.1%), followed by a gradual decline in prevalence at 20.2%, 9.0% and 3.6% in women who had 6–9, 2–5 and one, respectively (*p* for trend < 0.01). A similar trend was observed for HPV16/18, HPV16/18/31/33/45/52/58 and CIN2+ (*p* for trend < 0.01). In the fully adjusted model (model 3), the odds of having an HPV 16 or HPV 18 infection in women who had ≥ 6 lifetime sexual partners was 78-fold greater (*p* for trend < 0.01). For CIN2+ , the reference was 2–5 partners, since no woman with one partner had CIN2+ . Despite this, the odds of having CIN2+ in women who had 6–9 or ≥ 10 partners were still 2 to 4 times higher.

### Risk factors for high-grade cervical disease (Table 5)

**Table 5:** Risk factors for high-grade cervical disease (CIN2 or worse).

CIN2 or worse	n	(%pos)	Univariate			Multivariate		
			aOR	(95% CI)	*P* value	aOR	(95% CI)	*P* value
HPV type-specific infection								
Negative	7/2925	(0.2)	Reference		–	Reference		–
HPV16/18	13/76	(17.1)	126.1	(47.9–331.9)	< 0.01	113.7	(40.8–316.9)	< 0.01
Other high-risk HPV^a^	27/230	(11.7)	78.5	(33.6–183.5)	< 0.01	65.9	(27.1–160.2)	< 0.01
Age at first intercourse								
≤ 14	5/109	(4.6)	Reference		–	Reference		–
15–16	17/779	(2.2)	0.4	(0.2–1.2)	0.09	0.4	(0.1–1.4)	0.16
17–19	19/1441	(1.3)	0.3	(0.1–0.7)	0.01	0.4	(0.1–1.4)	0.15
≥ 20	6/902	(0.7)	0.1	(0.0–1.4)	< 0.01	0.4	(0.1–1.7)	0.19
Number of lifetime sexual partners								
1	0/526	(0.0)	0.0		0.99	0.0		0.99
2–5	16/1670	(1.0)	Reference		–	Reference		–
6–9	13/580	(2.2)	2.4	(1.1–5.0)	0.02	0.8	(0.4–1.9)	0.68
≥ 10	18/455	(4.0)	4.4	(2.2–8.8)	< 0.01	1.5	(0.7–3.6)	0.32

*CIN* cervical intraepithelial neoplasia, *aOR* adjusted odds ratio.

Adjusted for age, smoking history, age at first intercourse, number of lifetime sexual partners, HPV type-specific infection.

^a^HPV31/33/35/39/45/51/52/56/58/59/68.

Finally, we investigated risk factors for CIN2+ . Unlike HPV infection, lifetime number of sexual partners and age at sexual debut were not statistically significant factors for having CIN2+ when HPV genotype was adjusted for. Compared to women who were HPV negative women, women with a vaccine type HPV 16/18 lesion had a 114-fold risk for CIN2+ (*p* < 0.01), even after adjusting for sexual history, age and smoking history. The risk was 66-fold with a non-HPV 16/18 Hybrid Capture (HC)2 infection (*p* < 0.01).

## Discussion

There are four major steps in the development of cervical cancer: infection, persistence, progression and invasion. It is important to understand individual risk factors for each stage, as well as interventions to mitigate these risk factors in order to prevent them. Since there is no comprehensive data that combines all these risk factors in a Japanese population, we investigated the correlation between sexual behavior (age at sexual debut, number of sexual partners), HPV infection (including genotype) and high-grade cervical disease (CIN2+) in sexually active Japanese women of reproductive age. As a results, number of sexual partners was not an independent risk factor for CIN2+ when HPV genotype was adjusted for in the multivariate analyses. The greatest risk factor for CIN2+ was an HPV 16/18 infection (aOR 113.7).

Two previous studies have reported cohort differences in sexual behavior for Japanese populations^18,19^. One national study reported that 23% of female high school students (16–18 yrs) were sexually active in 2011 compared to only 9% in 1981. Similarly, in 2011, 46% of female university students (aged ≥ 18 yrs) reported being sexually active, compared to only 19% in 1981^18^. Regarding number of previous sexual partners, there is a paucity of robust Japanese data. There is only one national study on HIV by the MHLW in 1999 that reports such data. In this study, only the percentage of males and females by age-groups who had had ≥ 5 sexual partners is reported. Furthermore, the number of participants in each age-group is not given^19^. A more recent study by Imai et al. only reports numbers for university students^20^. In the present study, we found that 72% of women become sexually active as a teenager. Similar to the past national study, the age of sexual debut is becoming earlier in younger cohorts. In the present study of the general population, almost half of participants had 2–5 sexual partners and approximately 30% of participants had more than ≥ 6 sexual partners in their lifetime. There was a significant correlation between lifetime number of sexual partners and lower age at first intercourse.

Several cross-sectional studies have shown that early age at sexual debut is a risk factor for HPV infection^21^. In our univariate analysis we also found this to be true. However, after adjusting for number of sexual partners, we found that statistical significance disappeared.

Rate of acquisition of sexual partners, as well as the association between number of sexual partners and hrHPV infection is consistent and strong^7,22^. In the present study, we also found that even after adjusting for age, smoking history and age at first intercourse, the risk of a hrHPV infection significantly increased as the number of partners increased. For the two most oncogenic types, HPV 16 and HPV 18, risk of infection was 78-fold once the number of partners reached 6 or more. In the present study of women attending for screening, 32% of participants reported ≥ 6 sexual partners. In the national HIV study by Kihara et al., it was also reported that the younger one is, the likelihood of having ≥ 5 partners increases. (1.9% of women aged 55 yrs or older compared to almost 40% of women aged 18–24 yrs)^19^. While it was common in the past for women to marry at an early age, in 2016, the average age of marriage for Japanese women was 29.4 yrs, which means women could be in several monogamous relationship before choosing to settling down and that having more than one sexual partner does not reflect any particularly risky sexual behavior.

While many women may get cervical HPV infections, most infections do not persist or progress to high grade cervical disease. HPV genotype is not only the strongest viral cofactor, but also the strongest absolute risk for persistence and progression. HPV 16 is highly carcinogenic and responsible for over half (53.4%) of cervical cancers worldwide^23^. HPV 16/18 also have a faster time of progression to precancerous lesions^6^. Similar data have been reported for Japan^24^. In the present study, the greatest risk factor for CIN2+ was an HPV16/18 infection (aOR 113.7; *p* < 0.01), even after adjusting for sexual history, age and smoking history.

HPV vaccines have been available since 2006/2007. These vaccines induce a high-level antibody response to HPV 16 and HPV 18, responsible for over 70% of cervical cancers globally and over 90% of cervical cancers in Japanese women 20–29 yrs^23,25^. A recent systematic review of 10 year real-world use reported that HPV vaccines have dramatically decreased the incidence of vaccine-type HPV infection and high-grade cervical disease^26^.

Recent data from Japan has also shown vaccine effectiveness against HPV 16 and HPV 18 infections is extremely high (91.9%)^15^.Another study demonstrated that for cohorts eligible for free HPV vaccination, compared to those not eligible, the incidence of CIN2+ or CIN3+ in women attending cervical screening was significant lower, *p* = 0.014, *p* = 0.016^27^. However, the Japanese MHLW suspended proactive recommendations for the vaccine in June of 2013, and uptake plummeted from > 70% to,1%^16^.This means young Japanese women are once again at risk for HPV 16 and 18 related high-grade cervical disease^28^. This risk could be mitigated if the Japanese MHLW resumed proactive recommendations for the HPV vaccine. Furthermore, a recent modelling study has shown that rapid restoration of HPV vaccination coverage in girls aged 12 years, plus catch-up of those girls who missed vaccination due to the suspension of proactive recommendations using the nine-valent HPV vaccine, could mitigate much of the health impact of the HPV vaccine crisis in Japan to-date^29^. In the present study, the three most prevalent HPV types were HPV52, 16 and 58, which are covered by the nine-valent vaccine (supplementary Table1). In July 2020, the nine-valent HPV vaccine was finally approved for use in Japan, 5 years after filing for licensing^30^. Therefore, rapid recovery of vaccination coverage and widespread use of the nine-valent vaccine makes mitigation of much of the damage done so far possible.

For a comprehensive approach to cervical cancer prevention, education also plays an important role. The WHO states that “national educational campaigns for HPV vaccine introduction should be used to increase awareness about cervical cancer and its prevention”^31^. As part of Japan’s national cancer Control Plan, educational activities to increase school children’s awareness of factors to prevent cancers should be undertaken. To facilotate this, teaching materials were made available by the Ministry of Education, Culture, sports, Science and Technology^32^. However, with regards to cervical cancer prevention, only screening is recommended, no mention is given about the crucial role HPV infection plays in the etmology of cervical cancer and the fact that a highly safe and effective vaccine exists to prevent this infection is also omitted^17^. More education about HPV and the role it plays in the development of cervical cancer is urgently needed for young adolesents.

This study has several strengths. Firstly, it is one of the few studies to report sexual age at sexual debut and number of sexual partners in Japanese women. Secondly, to our knowledge, it is the only study to report the relationship between sexual behavior, type-specific HPV infection and high-grade disease status in a Japanese population. There are also several limitations that must be addressed. Firstly, our results may not reflect the population of Japan as a whole, since our study was only performed in one region of Japan, However, regarding age at sexual debut, our results were similar to a recent Japanese internet study on sexual behavior, conducted in all 47 prefectures of Japan^33^. A second limitation is that the number of women who had received the HPV vaccine was small (3.5%) which meant vaccination status could not be controlled for in the multivariate analyses. A third limitation is that due to a limited budget, the number of women that could be recruited in their thirties and forties was limited. Finally, our study has no information of male’s sexual behavior which is also a significant risk factor for HPV infection and cervical disease in women.

In conclusion, we found that > 30% of sexually active Japanese women had ≥ 6 previous sexual partners, that sexual debut is becoming earlier and associated with a greater number of sexual partners. We showed that the number of previous sexual partners is a significant risk factor for hrHPV infection, but HPV 16 and 18 infection and not number of sexual partners, is the most significant risk factor for CIN2+ . Educating adolescents about the HPV infection and achieving high uptake with the HPV vaccine, especially when given before the age of sexual debut would help mitigate these risk factors. Consequently, it is imperative that the Japanese government resumes proactive recommendations for the HPV vaccine and educates teenagers about the risks of HPV infection so that Japan women can be afforded the same level of protection against the disease as their global peers.

## Materials and methods

The cross-sectional study was conducted in Niigata city between April 2014 and March 2016. Niigata city has a population of around 790,000 and is the 16th most populous city in Japan. Women aged 20–30 yrs (group A), 35–36 yrs (group B) and 40–41 yrs (group C) were recruited when attending for public cervical cancer screening. Birth cohort years were 1984–1995, 1978–1980 and 1973–1975, respectively. Public funding for HPV vaccination started in 2010 for girls aged 13–16 yrs in Niigata city. Therefore, participants born after 1994 were eligible for free HPV vaccination. The present study protocol was approved by the institutional review board of Niigata University Graduate School of Medical and Dental Science and registered at the UMIN Clinical Trials Registry, trial number UMIN000030718. Written informed consent was obtained from all participants. All methods were performed in accordance with the relevant guidelines and regulations.

### Sexual history

Information about sexual history was obtained through a short self-administered questionnaire which asked about age at sexual debut and number of previous sexual partners. For the latter, participants had to choose from the following 5 categories: none, 1, 2–5, 6–9 and ≥ 10. Age at first intercourse was categorized into four groups: 14 yrs or younger, 15–16 yrs, 17–19 yrs and ≥ 20 yrs. Information on HPV vaccination status was also obtained from the questionnaire. As a potential confounder for persistence and progression of HPV infection, we also obtained information on smoking history from personal cervical cancer screening records. Responses were classified as: never, former or current smoker.

### HPV DNA testing

Residual liquid-based cytology (SURE PATH, BD Diagnostics, Sparks, MD USA) specimens from cervical screening were used for HPV testing. All samples were tested with HYBRID CAPTURE (HC) 2 (Qiagen, Hilden, Germany) for pooled infection with one or more 13 hrHPV genotypes; HPV types 16, 18, 31,33, 35, 39, 45, 51, 52, 56, 58, 59 and 68. Only those samples positive for the HC2™ test underwent HPV genotyping with the MEBGENTM HPV kit (MBL, Nagoya, Japan)^34^. This assay detects 13hrHPV genotypes; HPV types 16, 18, 31, 33, 35, 39, 45, 51, 52, 56, 58, 59 and 68.

### Cytologic and histologic diagnoses

Cervical cytology diagnoses were made at Niigata University and classified according to the Bethesda System^35^. Abnormal categories included cases of atypical squamous cells of undetermined significance (ASC-US) or worse. A result of negative for intraepithelial lesion or malignancy (NILM) was considered normal. All women with abnormal cytology, except for those cases that were ASC-US HPV negative, were referred for colposcopy. Information on cervical histology was obtained using data from Niigata city health center. For cervical histology, the three-tiered cervical intraepithelial neoplasia (CIN) classification (CIN 1, 2, and 3) was used. CIN2 or worse (CIN2+) was defined the high-grade cervical disease^36^.

### Statistical analyses

Characteristics of study participants were presented as means and SDs for continuous variables and as percentaged for categorical variables. Welch’s ANOVA test and polyserial correlation were used to investigate the correlation between age at first intercourse and number of sexual partners. Welch’s ANOVA test was also used to assess the difference in age at first intercourse between the three age-groups. Univariate and multivariate logistic regression analyses were conducted to investigate the correlation between sexual behavior (age at first intercourse and lifetime number of sexual partners), HPV infection status, HPV type-specific infection and cervical disease. Three models were used: model 1 adjusted for age; model 2 adjusted for age and smoking history; and model 3 adjusted for age, smoking history, age at first intercourse or number of sexual partners. Finally, we performed a multivariate analysis to investigate independent risk factors for high-grade cervical disease (CIN2+ vs. NILM). The model was adjusted for age, smoking history, sexual behavior, and HPV type-specific infection. We used IBM SPSS Statistics Version 25.0 (Chicago, USA) and R version 3.4.1 (Vienna, Austria). A two-sided *p* value of < 0.05 was considered to indicate statistically significant.

## Supplementary Information

Supplementary Information.
