# Supplementary material for: Risk factors for HPV infection and high-grade cervical disease in sexually active Japanese women
Source: Sci Rep. 2021 Feb 3;11:2898. doi: 10.1038/s41598-021-82354-6 (PMC7858628; doi:10.1038/s41598-021-82354-6)
Supplement: Supplementary file 1 — Supplementary Information. [file 41598_2021_82354_MOESM1_ESM.pdf]

Supplementary Information

Risk factors for HPV infection and high-grade cervical disease in sexually active Japanese women

Manako Yamaguchi, Masayuki Sekine, Sharon J. B. Hanley, Risa Kudo, Megumi Hara, Sosuke Adachi, Yutaka Ueda, Etsuko Miyagi and Takayuki Enomoto

Supplementary table 1. The prevalence of Human Papillomavirus (HPV) type-specific infection

	All (n=3231)	
	n	%
HPV infection		
HPV16	61	1·9%
HPV18	19	0·6%
HPV31	33	1·0%
HPV33	10	0·3%
HPV35	9	0·3%
HPV39	21	0·6%
HPV45	2	0·1%
HPV51	23	0·7%
HPV52	98	3·0%
HPV56	33	1·0%
HPV58	59	1·8%
HPV59	16	0·5%
HPV68	20	0·6%
